# Decoy nanoparticles protect against COVID-19 by concurrently adsorbing viruses and inflammatory cytokines

**DOI:** 10.1073/pnas.2014352117

**Published:** 2020-10-06

**Authors:** Lang Rao, Shuai Xia, Wei Xu, Rui Tian, Guocan Yu, Chenjian Gu, Pan Pan, Qian-Fang Meng, Xia Cai, Di Qu, Lu Lu, Youhua Xie, Shibo Jiang, Xiaoyuan Chen

**Affiliations:** ^a^Laboratory of Molecular Imaging and Nanomedicine, National Institute of Biomedical Imaging and Bioengineering, National Institutes of Health, Bethesda, MD 20892;; ^b^Biosafety Level 3 Laboratory, Key Laboratory of Medical Molecular Virology (Ministry of Education/National Health Commission/Chinese Academy of Medical Sciences), School of Basic Medical Sciences, Fudan University, 200032 Shanghai, China;; ^c^Institute of Medical Microbiology, Jinan University, 510632 Guangzhou, China;; ^d^State Key Laboratory of Virology, College of Life Sciences, Wuhan University, 430072 Wuhan, China;; ^e^School of Physics and Technology, Wuhan University, 430072 Wuhan, China

**Keywords:** COVID-19, SARS-CoV-2, cytokine storm, nanodecoy, cell membrane vesicle

## Abstract

The COVID-19 pandemic, caused by severe acute respiratory syndrome coronavirus 2 (SARS-CoV-2), has highlighted the urgent need to rapidly develop therapeutic strategies for such emerging viruses without effective vaccines or drugs. Here, we report a decoy nanoparticle against COVID-19 through a powerful two-step neutralization approach: virus neutralization in the first step followed by cytokine neutralization in the second step. The nanodecoy, made by fusing cellular membrane nanovesicles derived from human monocytes and genetically engineered cells stably expressing angiotensin converting enzyme II (ACE2) receptors, possesses an antigenic exterior the same as source cells. By competing with host cells for virus binding, these nanodecoys effectively protect host cells from the infection of pseudoviruses and authentic SARS-CoV-2. Moreover, relying on abundant cytokine receptors on the surface, the nanodecoys efficiently bind and neutralize inflammatory cytokines including interleukin 6 (IL-6) and granulocyte−macrophage colony-stimulating factor (GM-CSF), and significantly suppress immune disorder and lung injury in an acute pneumonia mouse model. Our work presents a simple, safe, and robust antiviral nanotechnology for ongoing COVID-19 and future potential epidemics.

## Significance

The COVID-19 pandemic caused by SARS-CoV-2 infection has led to more than 840,000 deaths worldwide as of August 31, 2020. Unfortunately, no licensed vaccine or specific treatment is available right now. Herein, we report a decoy nanoparticle against COVID-19. The decoy nanoparticles were constructed by fusing cell membrane nanovesicles derived from genetically engineered cells, which stably express SARS-CoV-2 receptor ACE2, and human monocytes, which display abundant cytokine receptors. By competing with host cells, these nanodecoys efficiently adsorb viruses and inflammatory cytokines such as IL-6 and GM-CSF. These two functionalities allow effective intervention of viral infection and its associated immune disorder, presenting a promising therapeutic strategy for COVID-19 and other potential epidemics.

COVID-19 pandemic, caused by a novel severe acute respiratory syndrome coronavirus 2 (SARS-CoV-2; also known as 2019-nCoV) (1), has resulted in more than 25 million infections and 840,000 deaths worldwide as of August 31, 2020 (2). Over the past 240 years, there have been several global epidemics caused by emerging and reemerging viruses, such as SARS-CoV, influenza A (H1N1) pdm09 virus, Zika virus, Ebola virus, and, most recently, SARS-CoV-2 (3). Each time, the lack of available drug or vaccine has greatly hindered effective protection against such an emerging viral threat (4). Thus, it remains a grand challenge and is of paramount importance to rapidly develop therapeutic strategies for ongoing COVID-19 or future potential epidemics.

Similar to SARS-CoV, the spike protein (S protein) of SARS-CoV-2 plays a vital role in viral infection. The S protein consists of S1 and S2 subunits: The S1 subunit engages human angiotensin converting enzyme II (ACE2) as the entry receptor, while the S2 subunit further facilitates viral fusion and entry (5–7). Responding to viral entry and infection, abundant inflammatory cytokines are up-regulated by macrophages/monocytes to eliminate pathogens and promote tissue repair (8). However, sustainably evaluated levels of inflammatory cytokines, characterized as cytokine release syndrome (CRS or “cytokine storm”), may in turn, exacerbate the inflammatory state and lead to immune dysfunction (9). Clinically, most patients with COVID-19 show mild symptoms, but ∼20% of patients progress to severe pneumonia, septic shock, and/or multiple organ failure owing to the CRS (10). Thus, in addition to vaccine development, approaches that block the viral entry involving ACE2 and treatments that suppress the aberrant inflammatory responses have become major focuses for COVID-19 (11).

Several antiviral drugs, including remdesivir, are being actively tested and have shown encouraging effects on the early intervention in SARS-CoV-2 infection (12, 13). However, there are very few drug candidates targeting late-stage infection-associated CRS. Interleukin-6 (IL-6), a proinflammatory cytokine, plays a pivotal role in many immunological diseases (14), and granulocyte−macrophage colony-stimulating factor (GM-CSF) is a myelopoietic growth factor involved in immune regulation (15). Several preclinical and clinical studies have reported that monoclonal antibodies targeting IL-6 and GM-CSF may potentially curb immunopathology caused by SARS-CoV-2 (16, 17), while it remains challenging to suppress CRS owing to the multiplicity of cytokine targets and the complexity of cytokine interactions (18).

Recent advances in nanotechnology and materials science, especially in lipid nanoparticles, offer many promising opportunities for infectious diseases (19–25). For instance, engineered liposomes, cell membrane nanosponges, and exosomes have been demonstrated to bind and neutralize bacterial toxin (19, 20, 26). Additionally, we have recently shown that biomimetic synthetic strategies involving synchronous synthesis and display of proteins on cell surface enable efficient development of cellular nanovesicles displaying proteins with native orientation, structure, and activity (22, 25, 27). Therefore, we hypothesize that we can genetically engineer ACE2 on cell surface and efficiently produce cellular nanovesicles displaying ACE2 to compete with host cells for SARS-CoV-2 binding (28–30). More importantly, recent reports involving cell membrane-coated nanoparticles for neutralization of broad-spectrum cytokines further promise the employment of engineered cellular nanovesicles for COVID-19 (18, 31).

**Fig. 1.**
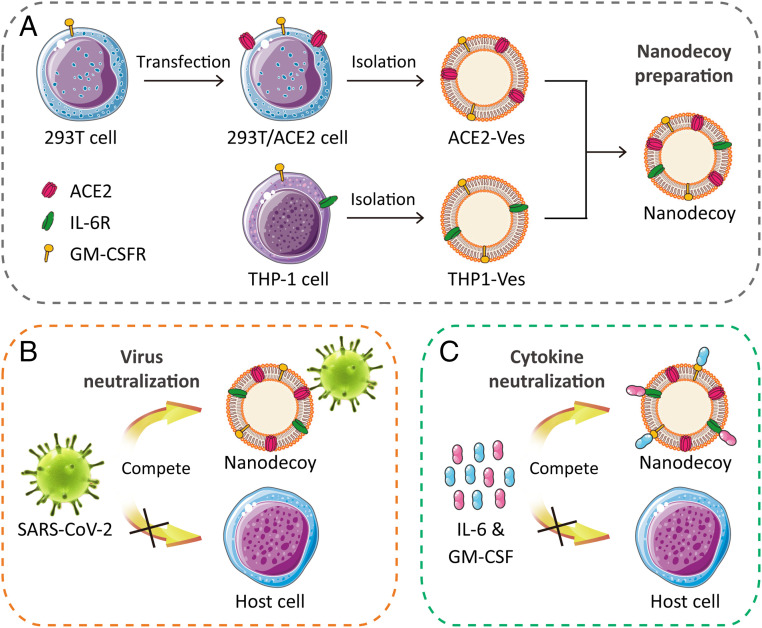
Schematic illustration of nanodecoys against COVID-19. (*A*) Preparation of nanodecoys by fusing cellular membrane nanovesicles derived from genetically edited 293T/ACE2 and THP-1 cells. The nanodecoys, displaying abundant ACE2 and cytokine receptors, compete with host cells and protect them from COVID-19 by neutralizing (*B*) SARS-CoV-2 and (*C*) inflammatory cytokines, such as IL-6 and GM-CSF.

Here, we develop an engineered cell membrane nanodecoy for COVID-19. Briefly, the nanodecoys were established in three steps: 1) genetically engineering ACE2 on human embryonic kidney 293T cells, 2) collecting cell membrane nanovesicles from engineered 293T/ACE2 cells and human myeloid mononuclear THP-1 cells, and 3) fusing the resulting two nanovesicles (Fig. 1*A*). In this design, the nanodecoys inherit abundant ACE2 and cytokine receptors from the source cells, enabling effective intervention of COVID-19 by concurrently neutralizing viruses and inflammatory cytokines (Fig. 1 *B* and *C*).

## Results

### Preparation of Nanodecoys.

**Fig. 2.**
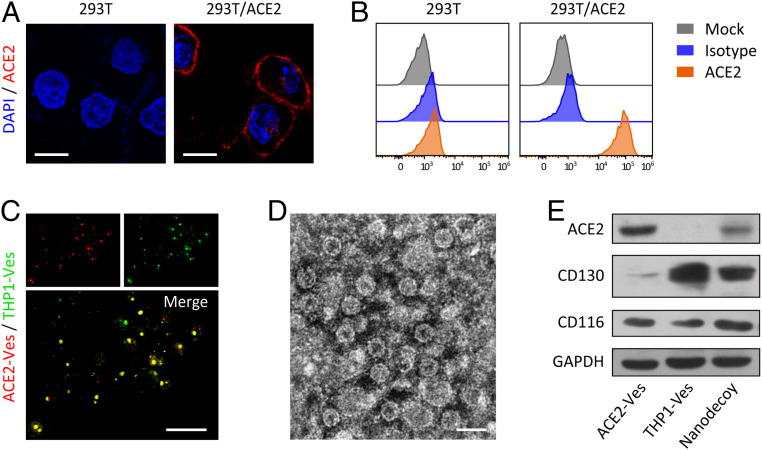
Preparation of nanodecoys. (*A*) Immunofluorescence imaging and (*B*) flow cytometry analysis of ACE2 expression on pristine and genetically engineered 293T cells. (Scale bars, 15 µm.) (*C*) Immunofluorescence images of nanodecoys. (Scale bar, 15 µm.) ACE2-Ves and THP1-Ves were labeled with different fluorescence dyes before fusion. (*D*) TEM image of nanodecoys. (Scale bar, 100 nm.) Samples were negatively stained with uranyl acetate. (*E*) Western blotting analysis of ACE2, IL-6 receptor CD130, and GM-CSF receptor CD116 in ACE2-Ves, THP1-Ves, and nanodecoys, respectively. GAPDH indicates glyceraldehyde-3-phosphate dehydrogenase.

Genetically engineered 293T/ACE2 cells were first prepared by transducing ACE2 onto 293T cells (32). Immunofluorescence imaging and flow cytometry confirmed the high expression of ACE2 on engineered cells (Fig. 2 *A* and *B*). To extract cell membrane nanovesicles, the intracellular content was removed by a combination treatment of hypotonic lysis, mechanical disruption, and gradient centrifugation (20). Subsequently, 293T/ACE2 and THP-1 cell membrane-derived nanovesicles (i.e., ACE2-Ves and THP1-Ves) were prepared by serial sonication and extrusion of cell membranes through nanopores on a mini extruder (20). After that, ACE2-Ves and THP1-Ves were mixed, sonicated, and repeatedly extruded through nanopores to form the nanodecoys (33). As a control, 293T cell-derived vesicles (i.e., 293T-Ves) were also prepared with similar procedures.

To determine whether different types of vesicles were indeed fused, ACE2-Ves and THP1-Ves were labeled with different fluorescent dyes before fusion. When the nanodecoys were viewed under a confocal microscope, significant overlap of fluorescence signals was observed (Fig. 2*C*), suggesting the fusion of ACE2-Ves and THP1-Ves. Subsequently, transmission electron microscopy (TEM) and dynamic light scattering (DLS) characterization revealed that the nanodecoys were round lipid droplets with an average size of 100 nm (Fig. 2*D* and *SI Appendix*, Fig. S1). Through Western blotting analysis, we confirmed that the nanodecoys preserved critical receptor proteins responsible for virus and cytokine binding, including ACE2 for SARS-CoV-2, CD130 for IL-6, and CD116 for GM-CSF (Fig. 2*E*). Furthermore, we quantified the ACE2 levels by enzyme-linked immunosorbent assay (ELISA) and demonstrated that 1 μg of nanodecoys contain ∼140 pg of ACE2.

### Nanodecoys Inhibit Pseudovirus and Authentic SARS-CoV-2 Infection.

**Fig. 3.**
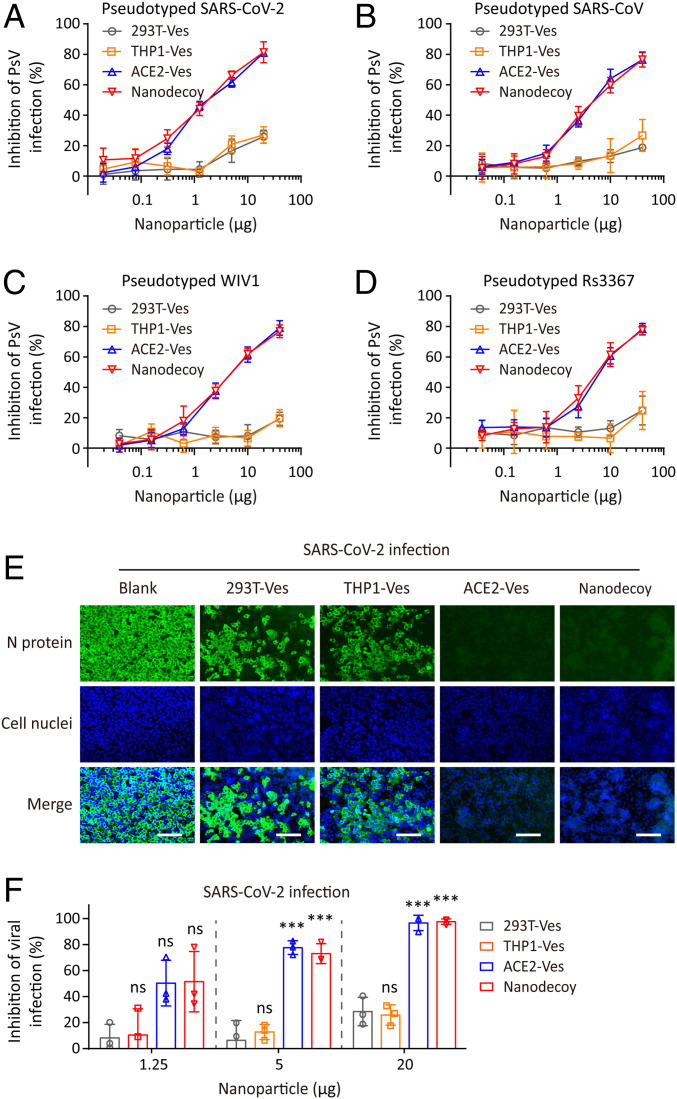
Nanodecoys inhibit pseudovirus and authentic SARS-CoV-2 infection. Inhibitory activity of nanodecoys against PsV (*A*) SARS-CoV-2, (*B*) SARS-CoV, (*C*) WIV1, and (*D*) Rs3367 infection. (*E*) Immunofluorescence images of SARS-CoV-2−infected Vero-E6 cells after treatment with nanodecoys. (Scale bars, 100 µm.) Cell nuclei and N protein of SARS-CoV-2 were labeled with DAPI (blue) and Alexa 488 (green), respectively. (*F*) Inhibitory activity of nanodecoys against SARS-CoV-2 infection on Vero-E6 cells. The nanodecoys used in this experiment contained an equal amount of ACE2 as compared with ACE2-Ves. Data points represent mean ± SD (*n* = 3). As compared with the 293T-Ves group, ns and *** indicate no statistical difference and *P* < 0.001, respectively.

After confirming the preparation of nanodecoys, we tested the antiviral effects of nanodecoys based on pseudoviruses (PsV). Pseudotyped SARS-CoV-2 was first packaged according to the protocols published previously (34), and human hepatoma Huh-7 cells were incubated with pseudotyped SARS-CoV-2 and nanoparticles (i.e., 293T-Ves, THP1-Ves, ACE2-Ves, or nanodecoys). We found that PsV infection was significantly inhibited by ACE2-Ves and nanodecoys, but not by 293T-Ves and THP1-Ves (Fig. 3*A*), suggesting that high-level ACE2 displayed on the surface of nanodecoys can hijack the S protein-mediated viral infection. SARS-CoV and SARS-related coronavirus (SARSr-CoV) also rely on human ACE2 receptor to gain the cell entry (34); therefore, we further tested the antiviral ability of nanodecoys based on pseudotyped SARS-CoV and SARSr-CoV, including strains of WIV1 and Rs3367. The nanodecoys exhibited potent inhibitory activity against pseudotyped SARS-CoV and SARSr-CoV in a manner similar to that of pseudotyped SARS-CoV-2 (Fig. 3 *B*–*D*), indicating the promising broad-spectrum antiviral capability of nanodecoys for SARSr-CoV, which may cause an outbreak similar to SARS-CoV-2 in the future.

Authentic SARS-CoV-2 was also used to test the antiviral ability of nanodecoys. To accomplish this, monkey kidney Vero-E6 cells were incubated with SARS-CoV-2 in the presence of nanodecoys and the expression level of viral nucleoprotein (N protein) was evaluated by immunofluorescence. Similar to PsV infection results, ACE2-Ves and nanodecoys showed better antiviral effects, as characterized by a reduction in viral particles (Fig. 3*E* and *SI Appendix*, Fig. S2). We further examined viral copy numbers in the supernatant with quantitative real-time polymerase chain reaction (qRT-PCR) (35), and demonstrated that the viral RNA level significantly decreased in a dose-dependent manner after treatment with nanodecoys (Fig. 3*F*). Meanwhile, the nanodecoys effectively inhibited pseudotyped and authentic SARS-CoV-2 infection on human colorectal adenocarcinoma epithelial Caco-2 cells (*SI Appendix*, Fig. S3), suggesting the potent antiviral effects of nanodecoys. Additionally, the cytotoxicity of nanodecoys was negligible (*SI Appendix*, Fig. S4), thus excluding the interference of cytotoxicity on the in vitro antiviral studies.

### Nanodecoys Neutralize Inflammatory Cytokines In Vitro.

**Fig. 4.**
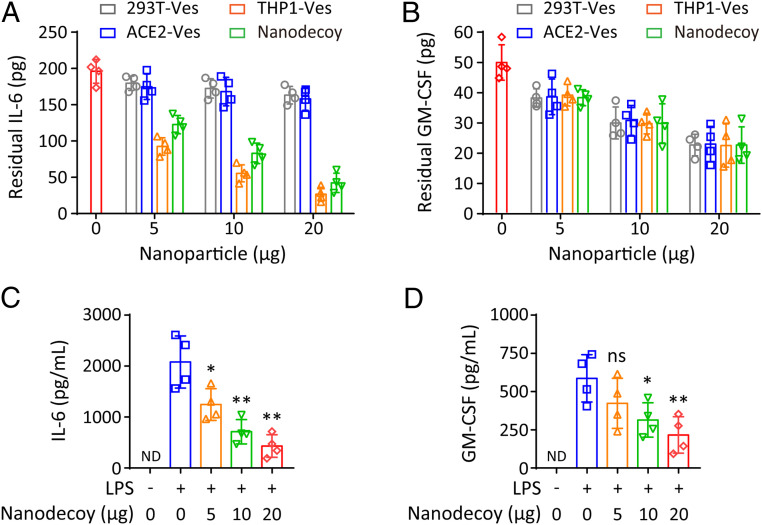
Nanodecoys bind and neutralize inflammatory cytokines. Removal of inflammatory cytokines including (*A*) IL-6 and (*B*) GM-CSF by nanodecoys. Inflammatory cytokines including (*C*) IL-6 and (*D*) GM-CSF in the supernatant of LPS-stimulated THP-1 cells after indicated treatments. ND indicates not detectable. Data points represent mean ± SD (*n* = 4). As compared with the group of LPS (+) and Nanodecoy (0), ns, *, **, and *** indicate no statistical difference, *P* < 0.05, *P* < 0.01, and *P* < 0.001, respectively.

The ability of nanodecoys to bind inflammatory cytokines was further investigated. Solutions containing known initial concentrations of IL-6 and GM-CSF were incubated with decoy nanoparticles. After the removal of nanoparticles, residual cytokines in the supernatant were quantified by ELISA (18). Benefiting from the abundant cytokine receptors on nanodecoys, we demonstrated that 20 μg of nanodecoys removed ∼160 pg of IL-6 and ∼25 pg of GM-CSF (Fig. 4 *A* and *B*), suggesting the promise of nanodecoys in effective suppression of cytokine storm. It should be noted that the cytokine removal ability of nanodecoys is dependent on many factors, including the receptor expression on nanodecoys and the affinity between cytokines and receptors; thus the nanodecoys showed varied capabilities on the removal of IL-6 and GM-CSF.

COVID-19 is closely associated with lung infection and injury, which may trigger the production of inflammatory cytokines, leading to enhanced recruitment of macrophages/monocytes, which, in turn, may exacerbate the inflammatory responses (8, 9). However, due to the lack of ACE2, THP-1 cells are not permissive for SARS-CoV-2 (36). To mimic the infection-induced inflammatory states, lipopolysaccharide (LPS) was used to stimulate THP-1 cells to up-regulate inflammatory cytokines (37). After the LPS treatment, both IL-6 and GM-CSF levels were significantly promoted (Fig. 4 *C* and *D*), confirming the activated inflammatory responses in this model. We then tested the cytokine neutralization performance of nanodecoys based on this model and found that the protein levels of IL-6 and GM-CSF significantly decreased after treatment with nanodecoys (Fig. 4 *C* and *D*), demonstrating effective intervention of activated inflammatory states by the nanodecoy treatment. While the current design involves IL-6 and GM-CSF, the cytokine neutralization capability of nanodecoys is also applicable to many other types of cytokines, because of the abundant cytokine receptors on the nanodecoy surface.

### Nanodecoys Suppress Acute Pneumonia In Vivo.

**Fig. 5.**
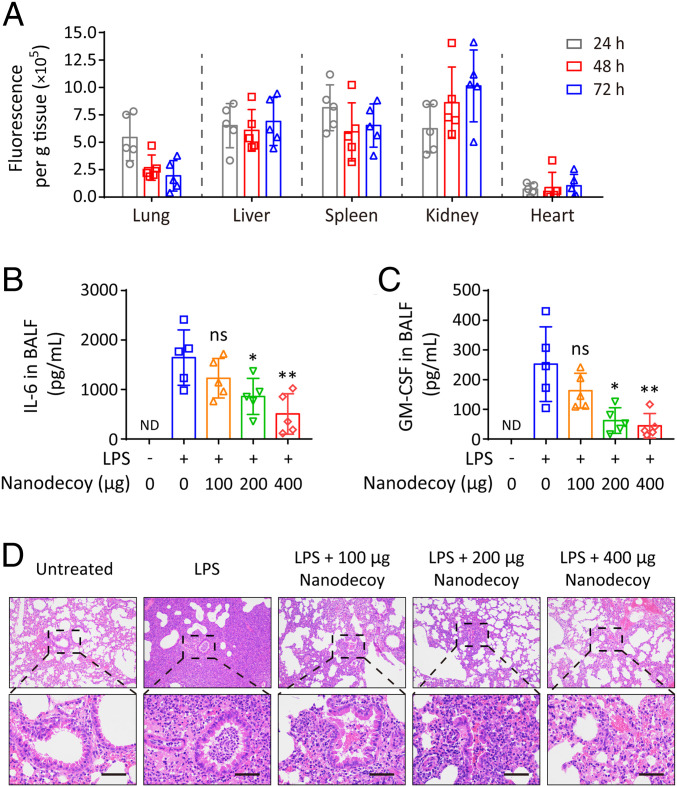
Nanodecoys suppress acute pneumonia in vivo. (*A*) Biodistribution of inhaled nanodecoys in major organs collected at indicated time points. Inflammatory factors including (*B*) IL-6 and (*C*) GM-CSF in the BALF after indicated treatments. ND indicates not detectable. (*D*) H&E-stained lung tissue sections after indicated treatments. (Scale bars, 50 µm.) Data points represent mean ± SD (*n* = 5). As compared with the group of LPS (+) and Nanodecoy (0), ns, *, and ** indicate no statistical difference, *P* < 0.05, and *P* < 0.01, respectively.

After confirming the cytokine neutralization ability of nanodecoys in vitro, we further tested the nanodecoys in vivo. Firstly, fluorescently labeled nanodecoys were administered to mice by inhalation, and the in vivo biodistribution of nanodecoys was studied (30, 38). A single dose of nanodecoys showed excellent retention in the lungs even after 72 h (Fig. 5*A*), indicating the potential of downstream inhalation delivery of nanodecoys for cytokine neutralization in vivo. Subsequently, we tested the in vivo performance of nanodecoys on an acute lung inflammation (ALI) mouse model. To induce ALI, the mice were first intratracheally (i.t.) inhaled with LPS (39). At 4 h post the challenge, the mice received i.t. inhalation of the indicated concentration of nanodecoys, and 8 h later, lung bronchoalveolar lavage fluid (BALF) was collected for cytokine measurement. In the BALF, IL-6 and GM-CSF levels were significantly higher after the LPS challenge (Fig. 5 *B* and *C*), demonstrating severe inflammatory status in the lung. Remarkably, the nanodecoys effectively decreased the IL-6, GM-CSF, and total protein levels in the BALF (Fig. 5 *B* and *C* and *SI Appendix*, Fig. S5), suggesting the encouraging effects of nanodecoys on broad-spectrum neutralization of inflammatory cytokines. Moreover, all mice were killed for histological examination at 24 h post the LPS challenge. Alveolar wall incrassation, alveolar cavity disappearance, vascular dilation and congestion, and alveolar inflammatory cell infiltration were observed in the lung of LPS-challenged mice (Fig. 5*D*), confirming the severe lung injury in this ALI model. Notably, the nanodecoys significantly suppressed the lung injury in a dose-dependent manner (Fig. 5*D*), suggesting the potential of nanodecoys in the inhibition of COVID-19-associated immune disorder and lung injury.

Systemic toxicity is a key issue for biomaterials (40). In this work, the mice were intravenously injected with phosphate-buffered saline (PBS) or PBS that contains nanodecoys every other day to investigate the in vivo toxicity. Neither death nor evident weight difference was observed between the PBS group and nanodecoy group (*SI Appendix*, Fig. S6). Serum biochemistry, complete blood test, and serum cytokine level examination were carried out at days 1, 7, and 15 post the first injection (*SI Appendix*, Figs. S7 and S8). Together with histology examination of major organs harvested at day 15 after treatment (*SI Appendix*, Fig. S9), we observed no significant side effects in mice. Although more detailed studies are necessary to further test short- and long-term systemic toxicity, our pilot toxicity study provides presumptive positive evidence supporting further development of these nanodecoys.

## Discussion

In summary, we have developed a decoy nanoparticle for COVID-19. By fusing the nanovesicles from genetically engineered cells and monocytes, the decoy nanoparticles, which display high-level ACE2 and abundant cytokine receptors, could compete with host cells for virus and cytokine binding. Based on pseudovirus and authentic SARS-CoV-2, we demonstrate that nanodecoys significantly inhibited viral replication and infection. Moreover, the nanodecoys efficiently bound and neutralized inflammatory cytokines, such as IL-6 and GM-CSF, and effectively suppressed the immune disorder and lung injury in an ALI mouse model. The synchronous neutralization of viruses and inflammatory cytokines allows effective protection against COVID-19.

The employment of engineered decoy nanoparticles against COVID-19 represents a promising antiviral nanotechnology with clear translational potential. However, further optimization and exploration are necessary. Scalability is always a key issue in the battle against such emerging pandemics. Mature gene editing, cellular vesicle purification, and fusion techniques promise rapid and large-scale production of nanodecoys. Another alluring feature of the nanodecoys is that the cellular vesicle components can be individually customized, providing a high degree of freedom in programmable development. Rare residual genetic materials in the nanodecoys may disturb host immunity, but short-term medication, together with evidence from our small-scale pilot safety study, could allay some of the safety concerns at this point in time. The host−virus affinity is also an important factor that needs to be accounted for. In the future, ACE2 variants with high affinity for viral S protein may further improve the blockade effects of nanodecoys on viral entry. Although detailed studies and further developments need to be carried out, our proof-of-concept work provides an approach to combat COVID-19 and other potential epidemics.

## Methods

### Cells.

Human myeloid mononuclear THP-1 cells, human primary embryonic kidney 293T cells, monkey kidney Vero-E6 cells, and human colorectal adenocarcinoma epithelial Caco-2 cells were obtained from the American Type Culture Collection. Human hepatoma Huh-7 cells were from the Cell Bank of the Chinese Academy of Sciences. Genetically transfected 293T/ACE2 cells were kindly provided by Dr. Lanying Du at Lindsley F. Kimball Research Institute, New York Blood Center, New York, NY. The cell lines were maintained in Dulbecco’s modified Eagle’s medium or Roswell Park Memorial Institute 1640 medium, supplemented with 10% fetal bovine serum (FBS), 100 U/mL penicillin, and 100 µg/mL streptomycin (all from Invitrogen).

### Virus Strain and Pseudotyped Viruses.

Patient-derived SARS-CoV-2 (nCoV-SH01) was isolated by Fudan University and used in this work. Various PsV particles were developed following the procedures below. The 293T cells were cotransfected with the HIV-1 backbone expressing luciferase reporters and one of the S protein expression vectors, including 293T/SARS-CoV-2/green fluorescent protein (GFP), 293T/SARS-CoV/GFP, and bat SARS-related CoV-S 293T/WIV1/GFP, or 293T/Rs3367/GFP. PsV was released in the supernatant, and the supernatant was collected at 72 h post the transfection, centrifuged at 3,000 × *g* for 10 min, and stored at −80 °C.

### Mice.

Adult Institute of Cancer Research (ICR) mice (20 g to 25 g, 4 wk to 6 wk old) were purchased from Hunan Silaike Jinda Laboratory Animal Co. Ltd. The animal study was approved by the Institutional Review Board of Wuhan University in accordance with the guidelines for the protection of animal subjects.

### Immunofluorescence.

To confirm the ACE2 on engineered 293T cells, parental and engineered 293T cells were plated into glass-bottomed dishes. After overnight culture, the cells were incubated with 1 μg/mL SARS-CoV-2 spike receptor-binding domain (RBD)-Fc recombinant proteins (Sino Biological) at 25 °C for 30 min. The cells were then stained with phycoerythrin (PE)-conjugated donkey anti-human IgG (Jackson ImmunoResearch) at 4 °C for 20 min. After being stained with DAPI, the cells were finally observed under confocal laser scanning microscopy (CLSM; ZEISS LSM700).

### Flow Cytometry.

Parental and ACE2-engineered 293T cells were incubated with 1 μg/mL SARS-CoV-2 spike RBD-Fc recombinant proteins for 1 h at 4 °C. Then PE-conjugated donkey anti-human IgG was used as a secondary antibody to stain the cells under 4 °C for 30 min; 7-AAD Viability Staining solution was employed to exclude the dead cells. Data were collected on a CytoFLEX flow cytometer and analyzed by using a matched CytExpert software (Beckman Coulter).

### Preparation of Nanodecoys.

THP-1 and 293T/ACE2 cells were suspended in hypotonic lysing buffer and disrupted by a Dounce homogenizer. The solution was treated with DNase and RNase (Invitrogen), and then centrifuged at 3,200 × *g* for 5 min. The supernatants were harvested and further centrifuged at 20,000 × *g* for 30 min, after which the supernatant was centrifuged again at 80,000 × *g* for 1.5 h. The pellets were collected, washed with PBS supplemented with protease inhibitor tablets, sonicated for 5 min, and finally extruded through polycarbonate membranes with 400-, 200-, and 100-nm pores on a mini extruder (Avanti Polar Lipids) to form cell membrane-derived nanovesicles (i.e., ACE2-Ves and THP1-Ves). The protein concentration of nanovesicles was measured by using a Bradford reagent (Sigma-Aldrich). ACE2-Ves and THP1-Ves were mixed (protein weight ratio of 1:1), sonicated for 5 min, and then extruded through 100-nm pores on the mini extruder. Anti-ACE2−modified immunomagnetic beads were designed, and pull-down assay was employed to purify the nanodecoys.

### Physicochemical Characterization of Nanodecoys.

The preparation of nanodecoys was monitored by measuring the zeta potential and hydrodynamic diameter with DLS (Nano-Zen 3600, Malvern Instruments). The morphologies of nanodecoys were also observed by TEM (JEM-2010HT, JEOL). Before TEM characterization, samples were prepared by contacting the droplet that contains nanodecoys with copper grids for 60 s followed by negatively staining with uranyl acetate for 30 s. Immunofluorescence imaging was also used to determine whether different types of vesicles were fused. Before membrane fusion, ACE2-Ves and THP1-Ves were labeled with 1,1′-dioctadecyl-3,3,3′,3′-tetramethylindodicarbocyanine, 4-chlorobenzenesulfonate salt (DiD; Thermo Fisher) and 1,1′-dioctadecyl-3,3,3′,3′-tetramethylindotricarbocyanine iodide (DiR; Thermo Fisher), respectively. After fusion, the nanodecoys were immobilized in glycerol and observed under CLSM. The concentration of ACE2 on nanodecoys was measured by an ACE2 ELISA Kit according to the instructions (Cloud-Clone).

### Western Blotting.

Samples containing ACE2-Ves, THP1-Ves, and nanodecoys were denatured and loaded into a 10% polyacrylamide gel. The proteins were then transferred onto polyvinylidene fluoride (PVDF) membranes, blocked with milk at 25 °C for 1 h, and incubated with primary antibodies: ACE2, CD130, and CD116 (all from AbClone) at 4 °C overnight. Finally, the PVDF membranes were further incubated with horseradish peroxidase-conjugated secondary antibody (Thermo Fisher), and the blots were developed by a West Pico PLUS Chemiluminescent Substrate Kit (Thermo Fisher).

### Inhibition of Pseudotyped Virus Infection.

Prior to infection, 50 μL of PsV was incubated with 50 μL of culture media containing the indicated concentration of 293T-Ves, THP1-Ves, ACE2-Ves, and nanodecoys (the nanodecoys used in this experiment contained an equal amount of ACE2 as compared with ACE2-Ves) at 37 °C for 45 min. Then 100 μL of the mixture was then transferred to target cells (Huh-7 and Caco-2 cells for SARS-CoV-2, and 293T/ACE2 cells for SARS-CoV and SARSr-CoV strains WIV1 and Rs3367), and culture media were changed after 12 h. After an additional 48 h of incubation, the Luciferase Assay System (Promega) was employed to analyze the luciferase activity.

### Inhibition of Authentic SARS-CoV-2 Infection.

Experiments involving SARS-CoV-2 were performed in a biosafety level 3 facility at Fudan University. Briefly, 50 μL of culture media containing the indicated concentration of 293T-Ves, THP1-Ves, ACE2-Ves, or nanodecoys (the nanodecoys used in this experiment contained an equal amount of ACE2 as compared with ACE2-Ves) was mixed with 50 μL of SARS-CoV-2 (750 pfu/mL) for 45 min and then added to Vero-E6 or Caco-2 cells. After adsorption at 37 °C for 1 h, the supernatant was replaced with fresh culture media with 2% FBS. After an additional 48 h, the supernatant was harvested, and the plates were fixed and stained.

### Viral Nucleoprotein Immunofluorescence.

After incubation with 100 μL of the mixture of SARS-CoV-2 and nanodecoy for 45 min, Vero-E6 cells were cultured for additional 48 h, washed with PBS, and fixed with 4% paraformaldehyde. After washing again, the cells were permeabilized with 0.2% Triton X-100, washed once more, and finally blocked with 3% bovine serum albumin for 1 h. Then, the cells were incubated with the SARS-CoV-2 nucleocapsid antibody (1:1,000; Thermo Fisher) at 4 °C overnight, followed by washing and incubating with Alexa Fluor 488-labeled goat anti-rabbit IgG (1:1,000; Thermo Fisher) for 1 h. After that, the cells were washed, incubated with DAPI for 5 min, washed again, and finally observed under fluorescence microscopy.

### Viral Gene Copies.

The qRT-PCR was employed to evaluate the RNA levels during SARS-CoV-2 infection. Viral RNA was extracted from the harvested supernatants by EasyPureViral DNA and RNA Kit (TransGen) and then measured by One Step PrimeScrip RT-PCR Kit (Takara).

### Inflammatory Cytokine Neutralization.

To determine nanodecoy binding with cytokines, 100 μL of PBS containing different concentrations of 293T-Ves, THP1-Ves, ACE2-Ves, or nanodecoys was mixed with 100 μL of PBS containing IL-6 (2,000 pg/mL) or GM-CSF (500 pg/mL), and incubated for 30 min. The samples were then centrifuged at 15,000 × *g* for 15 min to remove the nanoparticles, and the concentrations of IL-6 or GM-CSF in the supernatant were measured by IL-6 or GM-CSF ELISA Kit according to the manufacturer’s instructions (eBioscience).

### Suppression of LPS-Stimulated Inflammatory Responses In Vitro.

THP-1 cells were first cultured with 100 ng/mL of LPS for 24 h, and the indicated concentration of nanodecoys was added and incubated for additional 30 min. The supernatants were then collected and centrifuged at 15,000 × *g* for 15 min to remove the nanodecoys. The IL-6 or GM-CSF in the supernatant was measured by ELISA as described previously.

### Biodistribution of Nanodecoys after Inhalation Delivery.

Fifty microliters of Hank’s balanced salt solution (HBSS) containing 200 μg of DiD-labeled nanodecoys was i.t. delivered into the adult ICR mice via inhalation treatment with the MicroSprayer Aerosolizer and FMJ-250 High-Pressure Syringe Assembly (PennCentury). At 24, 48, and 72 h post the inhalation, the mice were killed, and major organs were carefully harvested and weighted for ex vivo fluorescence analysis with an in vivo imaging system (Perkin-Elmer).

### Inhibition of Acute Pneumonia In Vivo.

The mice were anesthetized and placed in a supine posture. Then 50 μL of HBSS containing 8 mg/kg of LPS from *Escherichia coli* (Sigma-Aldrich) was nebulized into the pulmonary alveoli of mice by using the Aerosolizer Assembly. At 4 h post the challenge, the mice received i.t. inhalation of 50 μL of HBSS or HBSS containing the indicated concentration of nanodecoys (i.e., 100, 200, and 400 μg). At 8 h after the nanodecoy injection, lung lavage was carried out by introducing 0.5 mL of HBSS into the lungs, and BALF was carefully withdrawn by inserting a needle into the upper trachea. The BALF was centrifuged at 15,000 × *g* for 15 min to pellet the nanodecoys. The IL-6 and GM-CSF in the BALF supernatant were determined by IL-6 and GM-CSF ELISA Kit (eBioscience) according to manufacturer’s guidelines. The total proteins in the BALF supernatant were measured by a Bradford reagent (Sigma-Aldrich). At 24 h post the LPS challenge, all mice were killed, and the lungs were routinely collected, fixed in 4% neutral buffered formalin, processed into paraffin, and sectioned at 4 μm. The sections were stained with hematoxylin and eosin (H&E) and observed under an optical microscope.

### Statistical Analysis.

All results are presented as mean ± SD. Statistical analyses were performed on GraphPad Prism 5.0 software. ANOVA test was used to analyze the significance of difference among four groups, and *P* values between each group were adjusted by Bonferroni correction; *, **, and *** indicate *P* < 0.05, *P* < 0.01, and *P* < 0.001, respectively.

## Supplementary Material

Supplementary File

## Data Availability

All study data are included in the article and *SI Appendix*.
