# Supplementary material for: Decoy nanoparticles protect against COVID-19 by concurrently adsorbing viruses and inflammatory cytokines
Source: Proc Natl Acad Sci U S A. 2020 Oct 6;117(44):27141–7. doi: 10.1073/pnas.2014352117 (PMC7959535; doi:10.1073/pnas.2014352117)
Supplement: Supplementary File [file pnas.2014352117.sapp.pdf]

PNAS
www.pnas.org

Supplementary Information for

Decoy nanoparticles protect against COVID-19 by concurrently adsorbing viruses and inflammatory cytokines

Lang Rao[a,1], Shuai Xia[b,1], Wei Xu[b,1], Rui Tian[a], Guocan Yu[a], Chenjian Gu[b], Pan Pan[c,d], Qian-Fang Meng[e], Xia Cai[b], Di Qu[b], Lu Lu[b,2], Youhua Xie[b,2], Shibo Jiang[b,2], and Xiaoyuan Chen[a,2]

[a] Laboratory of Molecular Imaging and Nanomedicine (LOMIN), National Institute of Biomedical Imaging and Bioengineering (NIBIB), National Institutes of Health (NIH), Bethesda, MD 20892, USA.
[b] Biosafety Level 3 Laboratory, Key Laboratory of Medical Molecular Virology (MOE/NHC/CAMS), School of Basic Medical Sciences, Fudan University, Shanghai 200032, China.
[c] Institute of Medical Microbiology, Jinan University, Guangzhou 510632, China.
[d] State Key Laboratory of Virology, College of Life Sciences, Wuhan University, Wuhan 430072, China.
[e] School of Physics and Technology, Wuhan University, Wuhan 430072, China.
[1] These authors contributed equally to this work.
[2] Corresponding e-mail: lul@fudan.edu.cn (L.L.); yhxie@fudan.edu.cn (Y.X.); shibojiang@fudan.edu.cn (S.J.); chen9647@gmail.com (X.C.).

This PDF file includes:

Supplementary Methods
Figures S1 to S9

www.pnas.org/cgi/doi/10.1073/pnas.2014352117

Supplementary Methods

Cytotoxicity assay

Cytotoxicity of nanodecoys to Huh-7 and Vero-E6 cells was tested by Cell Counting Kit-8 (CCK-8). Indicated cell type was seeded in 96-well plates and cultured at 37 °C for 12 h. After replacing medium containing indicated concentrations of nanoparticles, cells were further cultured at 37 °C for 48 h. The cells treated without any particles were used as a negative control. Before the test, CCK-8 solution was added, followed by an additional incubation for 4 h.

In vivo toxicity evaluation

ICR mice received an i.v. injection of 100 μL of PBS or PBS containing nanodecoys (200 μg per mouse) every other day. General status and body weight of the mice was evaluated at indicated time points. Complete blood panel test and serum biochemistry assay were conducted at days 1, 7 and 15 post first injection. Also, TNF-α and IL-6 levels in the serum was measured by mouse TNF-α and IL-6 ELISA Kit (eBioscience), respectively. All mice were euthanized at day 15 post first injection and the major organs were routinely collected, fixed in 4% neutral buffered formalin, processed into paraffin and sectioned at 4 μm. The sections were stained with hematoxylin and eosin (H&E) and observed under an optical microscope.

Supplementary Figures

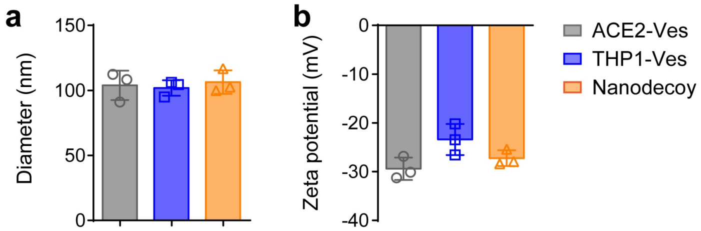

Figure S1. Preparation and characterization of nanodecoys. (**a**) Mean diameter and (**b**) zeta potential of ACE2-Ves, THP1-Ves, and nanodecoy. Data points represent as mean ± S.D. (*n* = 3).

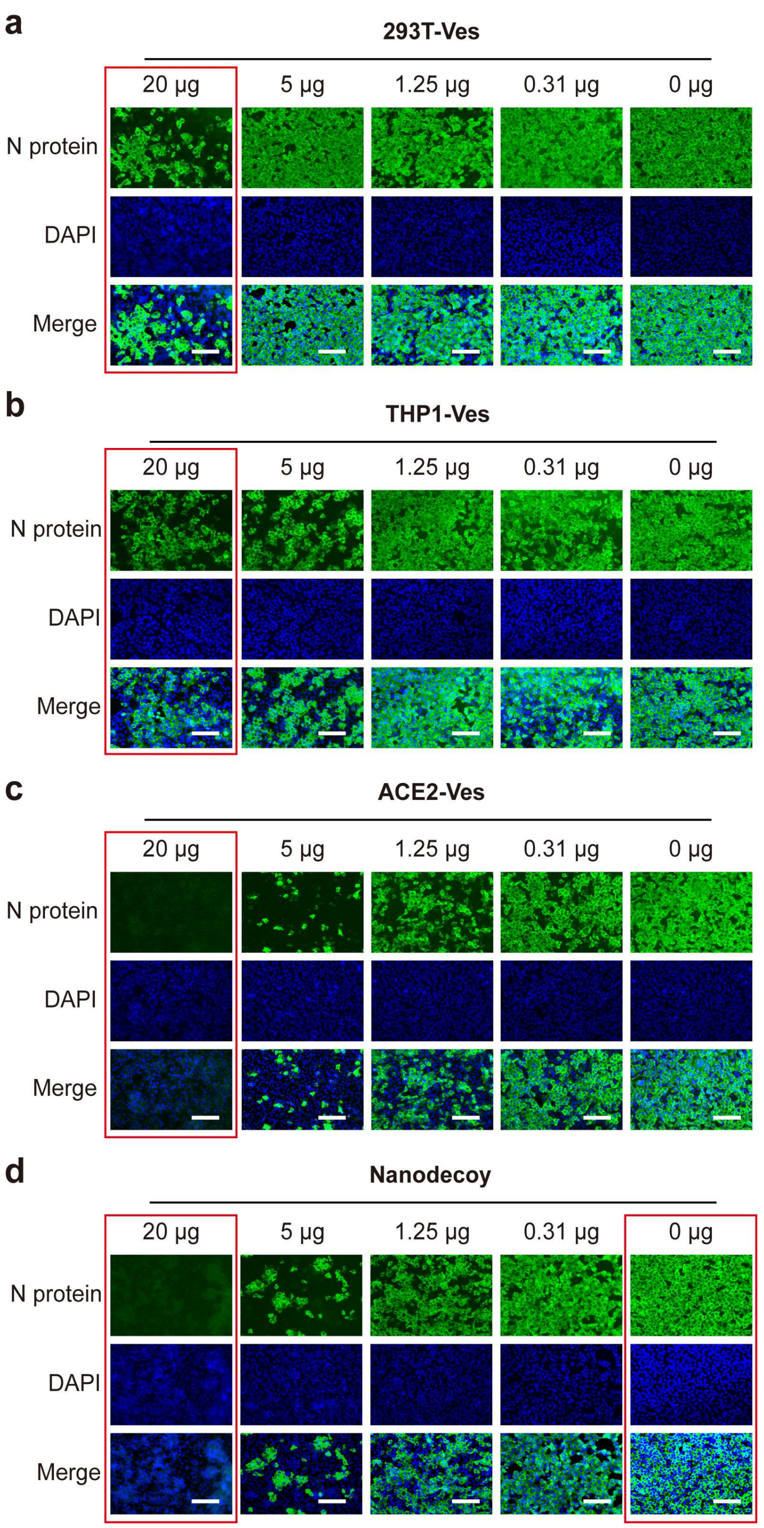

Figure S2. Immunofluorescence images of SARS-CoV-2-infected Vero-E6 cells after treatment with different concentrations of (**a**) 293T-Ves, (**b**) THP1-Ves, (**c**) ACE2-Ves, and (**d**) nanodecoy. Scale bars, 100 μm. Cell nuclei and the N protein of SARS-CoV-2 were labeled with DAPI (blue) and Alexa 488 (green), respectively. The images in the red boxes were rearranged and displayed in Figure 3e.

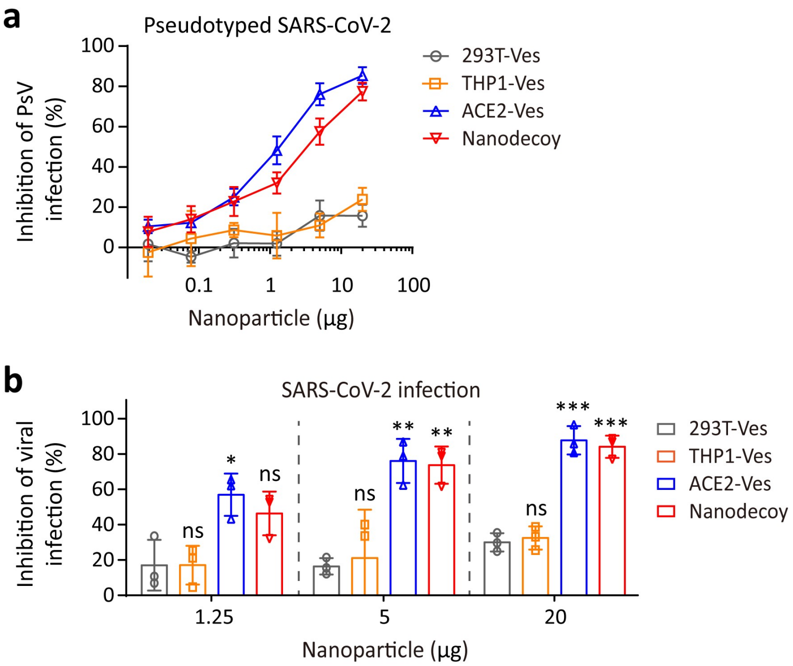

Figure S3. Inhibitory activity of nanodecoys against (**a**) pseudovirus (PsV) and (**b**) live SARS-CoV-2 infection based on Caco-2 cells. Data points represent as mean ± S.D. (n = 3). As compared with the 293T-Ves group, ^{ns}, *, **, and *** indicates no statistical difference, $P < 0.05$, $P < 0.01$, and $P < 0.001$, respectively.

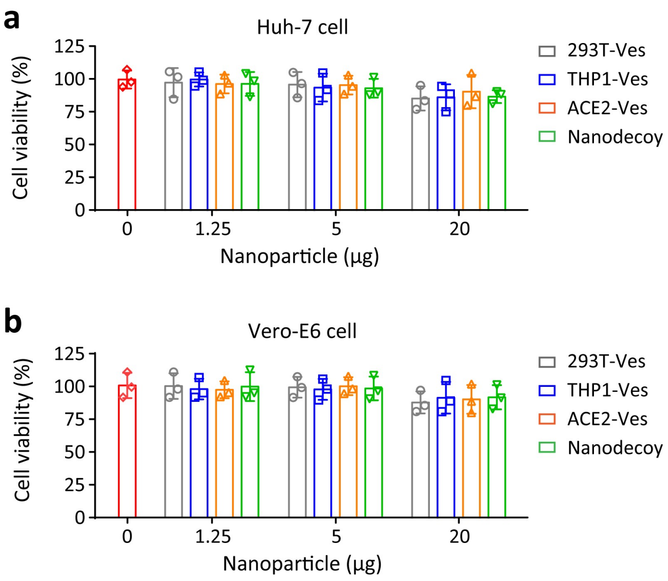

Figure S4. Viability of (**a**) Huh-7 and (**b**) Vero-E6 cells after treatment with different concentrations of nanoparticles. Data points represent as mean ± S.D. (n = 3).

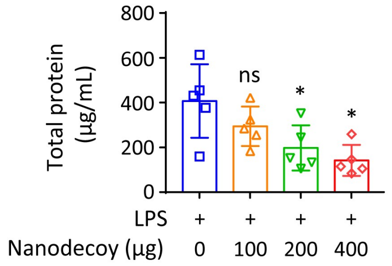

Figure S5. Total protein contents in BALF after indicated treatment. Data points represent as mean ± S.D. (n = 5). As compared with the group of LPS (+) and Nanodecoy (0), ns and * indicates no statistical difference and P < 0.05, respectively.

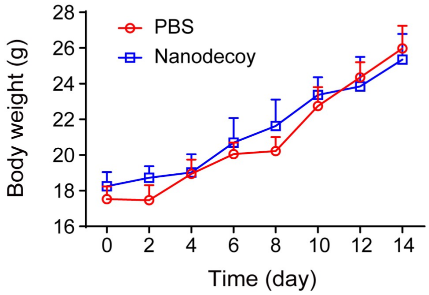

Figure S6. Mice body weight curves over 14 days. Data points represent as mean ± S.D. (*n* = 4).

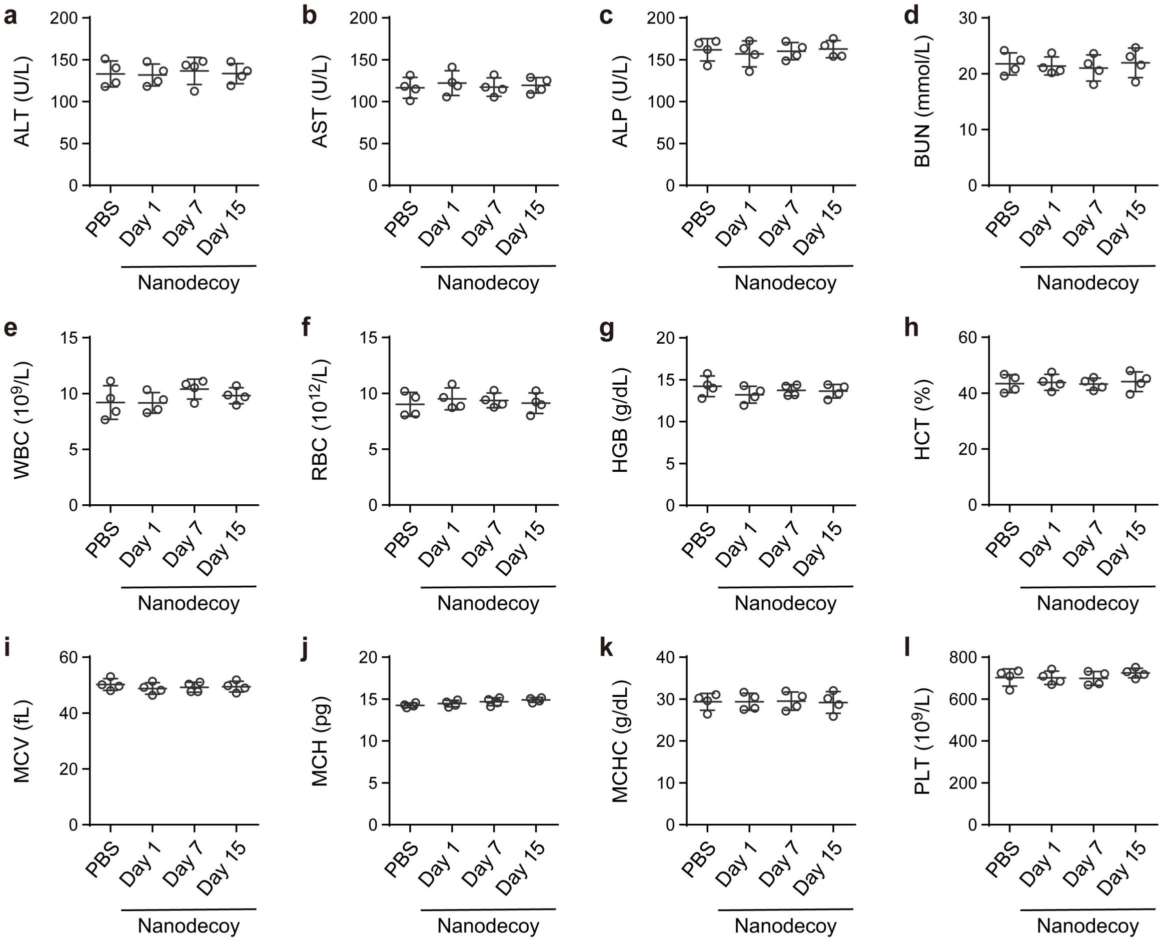

Figure S7. Blood biochemistry and complete blood tests. (**a**) ALT: alanine transaminase. (**b**) AST: aspartate aminotransferase. (**c**) ALP: alkaline phosphatase. (**d**) BUN: blood urea nitrogen. (**e**) WBC: white blood cell. (**f**) RBC: red blood cell. (**g**) HGB: hemoglobin. (**h**) HCT: hematocrit. (**i**) MCV: mean corpuscular volume. (**j**) MCH: mean corpuscular hemoglobin. (**k**) MCHC: mean corpuscular hemoglobin concentration. (**l**) PLT: platelets. Data points represent as mean ± S.D. (n = 4).

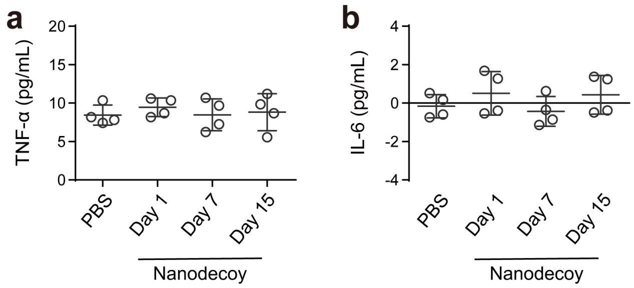

Figure S8. Inflammatory cytokine detection. (**a**) TNF-α and (**b**) IL-6 levels in the mouse sera as measured by ELISA. Data points represent as mean ± S.D. (n = 4).

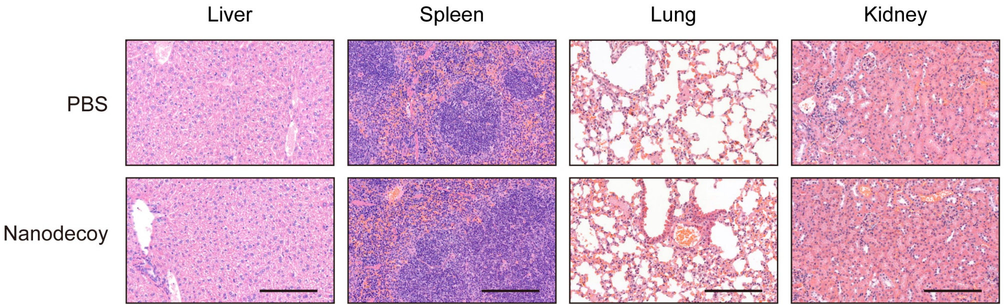

Figure S9. H&E-stained slice images of major organs. Scale bars, 200 μm.
